# Denosumab for Elderly Men with Osteoporosis: A Cost-Effectiveness Analysis from the US Payer Perspective

**DOI:** 10.1155/2015/627631

**Published:** 2015-12-09

**Authors:** Stuart Silverman, Irene Agodoa, Morgan Kruse, Anju Parthan, Eric Orwoll

**Affiliations:** ^1^Cedars-Sinai Medical Center, 8700 Beverly Boulevard, Los Angeles, CA 90048, USA; ^2^Amgen Inc., One Amgen Center Drive, Thousand Oaks, CA 91320, USA; ^3^Optum, 950 Winter Street, Suite 2800, Waltham, MA 02451, USA; ^4^Oregon Health & Science University, 3181 SW Sam Jackson Park Road, Portland, OR 97239, USA

## Abstract

*Purpose*. To evaluate the cost-effectiveness of denosumab versus other osteoporotic treatments in older men with osteoporosis from a US payer perspective. * Methods*. A lifetime cohort Markov model previously developed for postmenopausal osteoporosis (PMO) was used. Men in the model were 78 years old, with a BMD *T*-score of −2.12 and a vertebral fracture prevalence of 23%. During each 6-month Markov cycle, patients could have experienced a hip, vertebral or nonhip, nonvertebral (NHNV) osteoporotic fracture, remained in a nonfracture state, remained in a postfracture state, or died. Background fracture risks, mortality rates, persistence rates, health utilities, and medical and drug costs were derived from published sources. Previous PMO studies were used for drug efficacy in reducing fracture risk. Lifetime expected costs and quality-adjusted life-years (QALYs) were estimated for denosumab, generic alendronate, risedronate, ibandronate, teriparatide, and zoledronate.* Results*. Denosumab had an incremental cost-effectiveness ratio (ICER) of $16,888 compared to generic alendronate and dominated all other treatments. Results were most sensitive to changes in costs of denosumab and the relative risk of hip fracture.* Conclusion*. Despite a higher annual treatment cost compared to other medications, denosumab is cost-effective compared to other osteoporotic treatments in older osteoporotic US men.

## 1. Introduction

Worldwide, 1 in 5 men over the age of 50 is likely to have osteoporosis. Although osteoporosis is less common in men than women, 25–30% of all hip fractures are in men and the risk of mortality after osteoporotic fractures is greater in men than in women. The 10-year probability of major osteoporotic fractures increases with age and is particularly high in patients over 75 years old [1, 2]. Because of growth in the population of older people, the number of hip fractures is estimated to be 1.1 million in men by 2025 [3]. Osteoporotic fractures are associated with considerable morbidity and enormous health care costs [4]. In 2005, about 595,000 osteoporotic fractures were reported in US men, costing $4.1 billion [5].

Bisphosphonates and teriparatide are indicated for use in men with osteoporosis, and denosumab is indicated to increase BMD in men with osteoporosis at high risk for fracture [6]. Although denosumab is tolerable and efficacious in men, evidence suggesting economic value is also important. As the US healthcare system continues to evolve, US payers increasingly demand this type of economic information to support value driven decisions about therapeutic options. Denosumab has been demonstrated to be a cost-effective strategy compared to bisphosphonates and strontium ranelate in postmenopausal osteoporotic women in both Sweden and the US, as well in osteoporotic men in Sweden [7, 8]. Although denosumab was reported to be cost-effective in older osteoporotic men in Sweden, it is critical to evaluate the cost-effectiveness of denosumab in the US given the differences in the population characteristics, fracture rates, and healthcare reimbursement environment. Therefore, we evaluated the cost-effectiveness of denosumab in elderly osteoporotic men in the US and compared it to alendronate, risedronate, ibandronate, zoledronate, and teriparatide.

## 2. Methods

A previously published lifetime cohort Markov model was adapted for this analysis; the basic analysis methods and assumptions have been previously described [7, 8, 15]. Since fractures are more common in elderly men, the population of interest was men 75 years and older. The patient characteristics included in the model were reflective of a subgroup analysis of the more elderly participants enrolled in ADAMO (a multicenter, randomized, double-blind, placebo-controlled study to compare the efficacy and safety of denosumab 60 mg every six months versus placebo in males with osteoporosis), with mean age of 78 years, with a femoral neck BMD *T*-score of −2.12 and prevalent vertebral fractures in 23% [16, 17].

**Figure 1:**
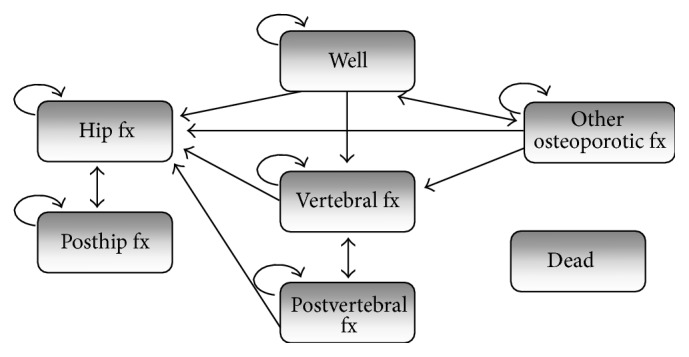
Model structure. Arrows to the health state “dead” are excluded for simplification.

Figure 1 illustrates the health states in the Markov model.

### 2.1. Treatment Efficacy

Since there is a paucity of well-powered trials reporting fracture risk reduction in osteoporotic men, data from postmenopausal osteoporosis (PMO) trials were used for the analyses (see Table 1). This approach was used because BMD improvements in response to interventions have consistently been shown to be similar across populations of osteoporotic men and women [16, 18–22]. For instance, in patients on denosumab who were ≥75 years old, the percentage change from baseline to month 12 in lumbar spine BMD was comparable between women in the FREEDOM trial (placebo: 0.7 (95% CI −0.1–1.4) versus denosumab: 4.8 (95% CI 4.1–5.6)) and men in the ADAMO trial (placebo: 1.0 (95% CI −0.5–2.5) versus denosumab: 4.8 (95% CI 3.3–6.4)) [16–18]. It is reasonable to assume that similar changes in BMD in men and women will reflect similar effects on fracture risk reduction. This assumption was substantiated in a recent clinical trial in which BMD change and vertebral fracture risk reduction from bisphosphonate therapy in men were similar to parallel studies in women [23]. Moreover, in recognition that therapy-induced fracture risk reduction is likely to be similar in men and women, regulatory agencies routinely approve osteoporosis treatments for fracture reduction in men when BMD improvements in men are similar to those in women [24]. In the absence of evidence for fracture reduction for a particular treatment at a particular skeletal site, 0% fracture risk reduction was assumed.

### 2.2. Treatment Duration and Persistence

In the model, patients received treatment up to 5 years except for teriparatide which is only indicated for 2 years of treatment.

The probability of treatment discontinuation within the first three years for the comparators was estimated using persistence data obtained from Weycker et al. and Landfeldt et al. [25, 26]. Persistence rates were based on a composite estimate of PMO patients taking oral bisphosphonates and then adjusted for men using the Landfeldt et al. data (Supplemental Table 1 in Supplementary Material available online at http://dx.doi.org/10.1155/2015/627631). The persistence rate for denosumab was estimated based on DAPS (Denosumab Adherence Preference Satisfaction) [27], which found patients on denosumab were 50% less likely to discontinue treatment (*P* = 0.029) than those given alendronate.

The other injectable osteoporosis treatments, teriparatide and zoledronic acid, were assumed to have the same persistence as denosumab (Supplemental Table 1).

### 2.3. Offset Time

Although antifracture efficacy is likely to persist for a period of time (offset time) after a treatment is stopped, there have been very few studies that report offset time and there is a lack of consensus on the duration of offset time [28–32]. The duration of offset time is likely to affect the number of incident fractures and mortality, and consequently costs and quality of life. Thus, in the current analyses, offset time was assumed to be equal to up to 2 years across all therapies in the base-case. However, given that some prior models [7] have used offset time up to 5 years for bisphosphonates, a sensitivity analysis was also conducted. In the sensitivity analyses, a 1- or 2-year offset times were applied for denosumab, up to 2.5 years for teriparatide, and up to 5 years for all other comparators.

Furthermore, in the model, it was assumed that offset time could not exceed the treatment duration. For example, if patients on oral bisphosphonates discontinued treatment by year 1, then their offset time was assumed to be 1 year. On the other hand, if patients on oral bisphosphonates received treatment for a full 5 years, then their offset time was assumed to be 2 years in the base-case and 5 years in the sensitivity analysis. Patients that drop out in the first 6-month Markov cycle did not receive any offset time.

### 2.4. Incidence of Fractures

The incidence of hip and clinical vertebral and other nonhip, nonvertebral (NHNV) osteoporotic fractures in untreated men were derived from Melton III et al. and Cooper et al. [33, 35] and are shown in Table 2. An explanation of the derivation methodology can be found in a previously published economic analysis [15]. In the absence of age-specific prevalence of morphometric vertebral fracture in the model, a constant risk of morphometric vertebral fracture was assumed when calculating the background population risk [34].

### 2.5. Mortality

Patients with osteoporotic fractures have a higher mortality compared to the general population [43]. In the model, the age-specific baseline mortality in the US normal population for men in 2010 was applied to the relative risk of mortality after a fracture in Swedish population [44] because US data were not available. It was assumed that mortality in the first year after fracture would be higher than in subsequent years for hip and vertebral fractures. NHNV osteoporotic fractures were assumed to only have an increased risk of mortality in the first year of fracture. The relative risk of mortality in men who have a fracture was estimated from Johnell et al. [43] and applied to the background mortality of the normal population. Relative risks of mortality related to fractures are shown in Supplemental Table 2 (for further methods detail, see Parthan et al.) [15].

### 2.6. Utility

The effect of hip fracture on quality of life, in the first year and subsequent years, was based on a meta-analysis [36]. The effect on quality of life from vertebral fracture in the first year was derived from Peasgood et al. [36]. It was assumed that the utility multiplier during the second and following years for a clinical vertebral fracture was 0.93 [37]. The disutility associated with NHNV fractures in the first year was derived from Borgström et al. 2006 [38]. The model assumed that the NHNV fractures did not have any impact on patients' quality of life in the second and subsequent years. The fracture-specific utility multipliers as shown in Table 3 were used together with the baseline utility values for normal US men [45].

### 2.7. Resource Use and Costs

The model included costs associated with the drug intervention, costs of treating fractures, drug administration, and monitoring costs and long-term care costs (see Table 4). All costs are reported in 2013 USD. Treatment costs including administration and monitoring were not applied for patients who discontinued treatment. Age-specific fracture costs by fracture site were derived from Brenneman et al. and Tosteson et al. [39, 40]. The Tosteson study includes costs for hip fracture patients in both men and women and was used to estimate the costs in the subsequent years following fracture. Costs associated with long-term care were considered in the model because many people with a hip fracture are discharged to a long-term care facility. Since there were no published data on cost of nursing home care in men, the costs were estimated using data from women. Patients with vertebral and NHNV fractures were assumed not to be associated with any long-term costs.

### 2.8. Analyses

The model was used to estimate both cost and outcomes over a lifetime horizon for each treatment strategy. Total costs included costs of treatment intervention (both drug and administration costs and osteoporosis management costs), direct medical costs for all fracture types, and long-term care costs of a nursing home (as a result of hip fracture). Total QALYs (quality-adjusted life-years) were calculated using the product of the utility weights for each health state and the time spent in that health state and summed for all health states over the patient's lifetime. Total LYs (life-years) were calculated by adding all the time the patients spent in a nondeath health state. Costs and health outcomes were discounted at 3% annually. Incremental cost-effectiveness ratios (ICERs) were reported as the cost per QALY gained and cost per LY saved. The model also reported the 10-year incidence of all fracture types for each treatment strategy.

As is the standard in health economic methodology, treatment strategies were rank ordered by increasing cost and ICERs were calculated successively for each next most costly strategy. Dominated strategies, those with both higher costs and lower efficacy than a comparator, were removed from the analysis.

### 2.9. Sensitivity Analyses

Parameters were varied using published confidence intervals or standard errors, where available, and by 25% above and below their base-case values when not available. The model was rerun while holding all other parameters fixed. Since zoledronate has lost exclusivity, the drug acquisition cost was reduced by 35% and 65% of the current price in sensitivity analyses. As risedronate is expected to lose exclusivity in 2014, a similar analysis was done using the estimated generic price of risedronate.

A probabilistic sensitivity analysis (PSA) was performed to assess uncertainty in the model. The PSA was performed by simultaneously drawing from appropriate distribution functions for each model parameter according to their means and standard errors. This process of drawing parameters and running the model was repeated 1,000 times and the results are presented graphically. The parameters included in the PSA were efficacy of denosumab and the comparators, costs of fractures, utilities, the DAPS ratio, and proportion of patients going to long-term care after hip fracture.

## 3. Results

### 3.1. Base-Case Results

Results of the multiway cost-effectiveness analysis show that generic alendronate had the lowest costs followed by denosumab (Table 5). Compared to generic alendronate, the lifetime costs associated with denosumab were approximately $900 higher per patient. However, men on denosumab had 0.05 additional QALYs per patient. This resulted in $16,900 per QALY gained. Denosumab had lower costs and higher QALYs per patient than all other comparators, which meant it “dominated” the other comparators.

**Figure 2:**
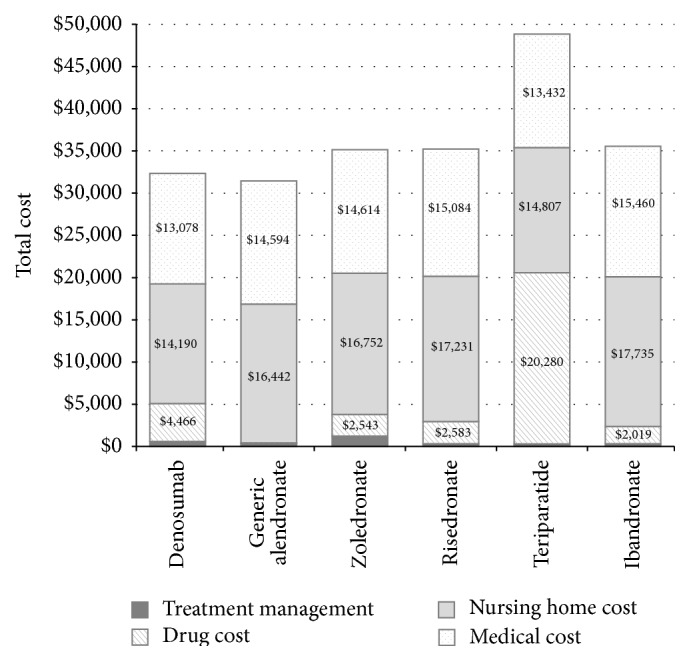
Base-case disaggregated costs.

Compared to all other therapies, patients on denosumab had the lowest 10-year risks of hip fractures in the model (Table 6).  Figure 2 displays the disaggregated costs. Across most treatment strategies, costs associated with long-term care accounted for the majority of the lifetime costs. The exception was that drug acquisition costs were the highest in teriparatide patients.

### 3.2. Sensitivity Analyses

**Figure 3:**
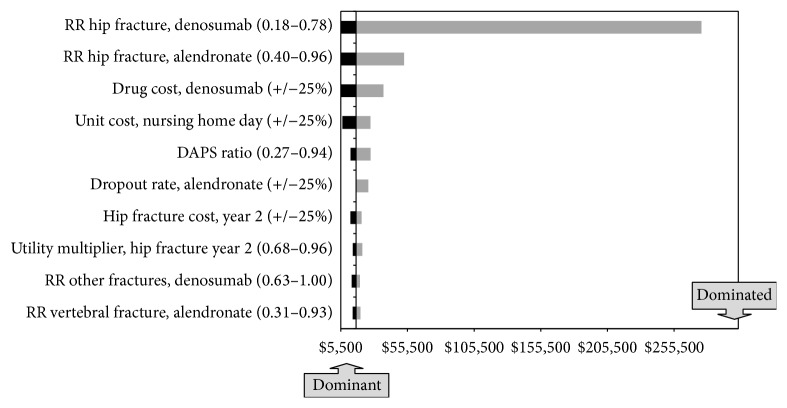
Base-case deterministic sensitivity analysis.

The ICER for denosumab versus generic alendronate is most sensitive to changes in the relative risk of hip fracture with denosumab. When this risk is lowered to 0.18, denosumab dominates generic alendronate. When this risk is increased to 0.78, the ICER for denosumab relative to alendronate is $276,100 per QALY gained. Other sensitive parameters include the relative risk of hip fracture with alendronate, the drug cost of denosumab, and the unit cost of a day in the nursing home (Figure 3).

Using an estimated generic price for zoledronate and risedronate (assumed to be 35% and 65% reductions of the base-case costs, resp.), results did not change; denosumab dominated zoledronate and risedronate. Similarly, results were mostly unchanged when offset times were varied; the ICER for denosumab compared to generic alendronate was $22,000 and denosumab dominated all other comparators. In sensitivity analyses, when the offset time for denosumab was reduced from 2 years to one year and all other comparators remained at 2 years, results were similar (ICER = $29,500).

**Figure 4:**
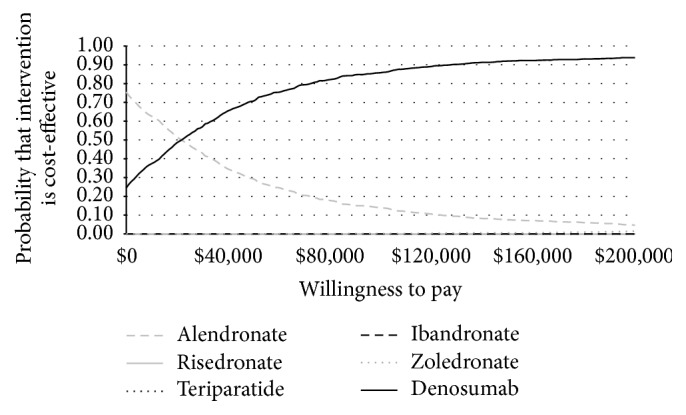
Base-case probabilistic sensitivity analysis. Note: CE curves for risedronate, ibandronate, and teriparatide do not appear in the figure, as they are not considered cost-effective at any threshold in this analysis.

The probability of denosumab being cost-effective compared to the other osteoporotic treatment strategies, including generic alendronate, at a threshold of $100,000 [46] per QALY was 85.8%. A cost-effectiveness acceptability curve for the probabilistic sensitivity analyses is illustrated in Figure 4.

## 4. Discussion

In this study, the cost-effectiveness of denosumab compared to other osteoporotic treatments was evaluated in men who were similar to the average elderly patient characteristics in the ADAMO trial. Denosumab had an ICER of $16,900 compared to generic alendronate and dominated all other treatment strategies included in the study. Compared to all other treatments, the probability of denosumab being cost-effective at a threshold of $100,000 [46] per QALY was 85.8%. Results were most sensitive to changes in the relative risk of hip fracture on denosumab, the relative risk of hip fracture with alendronate, the drug cost of denosumab, and the unit cost of a day in the nursing home. The economic benefits of denosumab are probably more pronounced in the elderly population, as hip and vertebral fractures are more common, leading to higher economic costs, morbidity, and mortality [9].

Denosumab was cost-effective using our base-case model. When assumptions and inputs were varied in sensitivity analyses to reflect areas of uncertainty, the results were basically unchanged. This indicates that the simple unit cost of a drug should be only one of several factors used in deciding the most appropriate therapy. Other considerations, such as persistence and efficacy across all fracture types, must be taken into account to recognize the full economic value.

With the exception of the cost-effectiveness study in elderly Swedish men [15], which used the Markov model structure used in the current analysis, we do not know any other published studies that evaluated the cost-effectiveness of different osteoporotic treatments in older men with osteoporosis. In the study of costs in Sweden, denosumab was compared to generic alendronate, generic risedronate, ibandronate, strontium ranelate, zoledronate, and teriparatide. Denosumab dominated all other comparators by having the lowest costs and the greatest QALYs.

This analysis of a cost-effectiveness model based in the US is important despite the previous report in the Swedish context. There are differences in population characteristic, generic availability, and healthcare costs between Sweden and the US that could influence cost-effectiveness estimates and suggest the need for independent analyses. Country-specific data were used for each model and included inputs of background population mortality, background population utility, and background population fracture risk, as well as direct medical costs of fractures, monitoring, and treatments. Persistence data were also country-specific. Due to the availability of data, patients in the US were only at risk of premature discontinuation for the first 3 years while Swedish patients were at risk for 4 years. Despite those differences, findings from the current study and the previous Swedish study are similar; in both, denosumab was a cost-effective treatment in older osteoporotic men. While the population fracture risk, treatment options, and discontinuation patterns differ across countries, denosumab is still the most effective treatment. These findings could potentially be extrapolated to other geographic settings as well.

The assumptions made in the model structure and model inputs are important. The cohort Markov model assumes a hierarchical structure; that is, once patients have experienced a fracture, they cannot have another milder fracture type. Patients from the posthip fracture state can either sustain another hip fracture, but they cannot experience a vertebral fracture or a NHNV osteoporotic fracture. Patients with vertebral fractures can only incur new vertebral fractures or hip fractures, but not NHNV osteoporotic fractures. Because of this hierarchical structure, the number of milder fractures in the cohort is likely to be slightly underestimated. The model also assumed that patients with vertebral and NHNV osteoporotic fractures do not incur costs beyond the first year of fracture; this may slightly underestimate fracture costs. The relative risks reported for nonvertebral fractures, which may include hip as well as NHNV fractures, were applied for NHNV fractures; this might slightly overestimate the number of NHNV fractures.

Published data concerning the rate of drug-specific discontinuation was only available for the first 3 years of treatment. In the current analysis (except for teriparatide), patients remaining on therapy after 3 years were assumed to continue until planned termination at 5 years. This assumption is supported by long-term studies of discontinuation rates of osteoporosis medications (considered as a group) indicating they are the highest shortly after the initiation of treatment, after which these rates remain stable for 5 or more years [47, 48]. Discontinuation rates for oral medications were taken from a registry study, but registry data were not available for injectable medications. Therefore, the model used a hazard ratio from the DAPS study [27] to calculate the discontinuation of denosumab and other injectable treatments. Using this methodology, our model predicts 73.6% of patients are persistent with denosumab at one year. This is consistent with two recent denosumab studies, one prospective and one retrospective, which found 82% persistence at one year and 70% persistence at eight months, respectively. Teriparatide is a daily injection, while denosumab and zoledronic acid are 6-month and annual injections, respectively. However, data reported by Landfeldt et al. [25] shows that 1-year persistence rates between denosumab and teriparatide are similar. Due to lack of additional data, we assumed the persistence rate for teriparatide to be equal to denosumab at 2 years as well. Although patients are likely to discontinue teriparatide treatment after 18 months, it is more conservative to assume persistence equal to denosumab at 2 years. Also, in applying the same HR to real-world data for all injectable treatments (denosumab, teriparatide, and zoledronic acid), the model minimizes the potential bias.

This analysis has several potential limitations. First, to most accurately model the effects of denosumab in men, the model's target population was reflective of the elderly men in the ADAMO trial, but it may not represent all male osteoporotic patients. Second, in the real-world setting, patients may receive sequential treatments such as receiving alendronate after discontinuing teriparatide. However, the current model does not take into account pretreated patients or sequential treatments. Nevertheless, the model structure used in the analyses has been widely used in osteoporosis cost-effectiveness research (Jönsson et al. 2011 [7], Borgström et al. 2006 [38], and Kanis et al. 2008 [49]). Only one study by Liu et al. 2006 [50], examined sequential treatment (using teriparatide followed by alendronate) and assumed that the reduction in the risk of fracture was equal to a patient that had not been pretreated. Third, it was assumed here that generic and branded alendronate would have comparable efficacy and safety data. However, Kanis et al. suggest evidence that generic alendronate may be less well tolerated than the branded alendronate. This may affect adherence poorly and thus lead to poorer fracture outcomes, which could impact the cost-effectiveness results [51]. Finally, in the absence of adequate efficacy data in treatment trials in osteoporotic men, fracture reduction rates for the base-case model were derived from studies of PMO. We believe this is reasonable because there is little theoretical reason to suspect treatments will have different effects in men and women, BMD improvements have been shown to be similar across trials in men and women, and the fracture reduction data that are available (e.g., Boonen et al. [23]) indicate similar effects regardless of sex.

The results from this economic analysis suggest that denosumab is a cost-effective option compared to other existing treatments for older osteoporotic men in the US. Even though alendronate is a low cost generic therapy, using a threshold of $100,000 per QALY, denosumab was cost-effective compared to generic alendronate. When the prices of zoledronate and risedronate were reduced by 35% and 65%, denosumab was still dominant. The differences in fracture risk reduction and improved persistence with denosumab are the largest drivers for denosumab being cost-effective compared to the other strategies. This analysis illustrates that the selection of treatment for men with osteoporosis should consider factors in addition to simple per dose costs. The significance of selecting the most appropriate osteoporosis treatment may be especially important for planning the overall costs per patient to a health care system payer.

## Supplementary Material

The supplementary material provides two tables with additional detail on model estimation. The first table provides 6-month discontinuation rates for all treatments, up to three years. The second table provides the relative risks of mortality for each fracture type, compared to the general population.

## Figures and Tables

**Table 1:** Relative risk of fractures.

	Hip	Vertebral	NHNV	Source
Denosumab	0.38	0.36	0.84	Boonen et al. 2011 [9], McClung et al. 2012 [10]
Generic Alendronate	0.62^*∗*^	0.62	0.82^*∗*^	NICE Evidence Review 2008 [11] Inderjeeth et al. 2009 [12]
Zoledronate	0.82	0.34	0.73	Boonen et al. 2010 [13]
Risedronate	0.85	0.56	0.80^*∗*^	McClung et al. 2001 [14] Inderjeeth et al. 2009 [12] NICE Evidence Review 2008 [11]
Ibandronate	1.00^*∗*^	0.51^*∗*^	1.00^*∗*^	NICE Evidence Review 2008 [11]
Teriparatide	0.25^*∗*^	0.35^*∗*^	0.47^*∗*^	NICE Evidence Review 2008 [11]

^*∗*^Where data are unavailable in the PMO elderly, the RRs are assumed to be similar to the overall PMO population.

**Table 2:** Incidence of fractures.

Age	Hip^*∗*^	NHNV^*∗*^	Morphometric vertebral^†^	Clinical vertebral^‡^
75–79	0.0053	0.0076	0.2	0.0045
80–84	0.0060	0.0203	0.2	0.0045
85+	0.0150	0.0291	0.2	0.0133

^*∗*^Source: Melton et al. 1999 [33].

^†^Source: Hasserius et al. 2003 [34]; value shown is prevalence.

^‡^Source: Cooper et al. 1992 [35].

**Table 3:** Utility multipliers by fracture type and adverse event.

Fracture type/period	Utility multiplier	Source
*First year after fracture*		
Hip fracture	0.700	Peasgood et al. 2009 [36]
Clinical vertebral fracture	0.590	Peasgood et al. 2009 [36]
NHNV fractures	0.902	Kanis et al. 2004 [37]
*The second year and following years after fracture*		
Hip fracture	0.800	Peasgood et al. 2009 [36]
Clinical vertebral fracture	0.930	Borgström et al. 2006 [38]

**Table 4:** Resource use and unit costs.

Resource	Cost	Frequency	Source
Hip fracture			
Year 1	$28,112	—	Brenneman et al. 2013 [39]
Year 2+	$9,734	—	Tosteson et al. 2008 [40]
Vertebral fracture			
Year 1	$7,882	—	Brenneman et al. 2013 [39]
NHNV fracture	$9,236	—	Brenneman et al. 2013 [39]
Nursing home (per day)	$236	—	Brenneman et al. 2013 [39]
BMD measurement	$243	Once every 2 years	Physician's Fee and Coding Guide 2013 [41]
Physician visit	$100	Once per year	Physician's Fee and Coding Guide 2013 [41]
Intravenous (IV) injection	$151	Once per year (zoledronate only)	Physician's Fee and Coding Guide 2013 [41]
Nurse visit	$42	Twice per year (denosumab only)	Physician's Fee and Coding Guide 2013 [41]
Denosumab (yearly)	$1,650	—	EncoderPro.com WAC 2013 [42]
Generic alendronate (yearly)	$30	—	EncoderPro.com WAC 2013 [42]
Zoledronate (yearly)	$1,084	—	EncoderPro.com WAC 2013 [42]
Risedronate (yearly)	$1,708	—	EncoderPro.com WAC 2013 [42]
Ibandronate (yearly)	$1,332	—	EncoderPro.com WAC 2013 [42]
Teriparatide (yearly)	$14,514	—	EncoderPro.com WAC 2013 [42]

All costs have been inflated to 2013 USD where necessary.

**Table 5:** Cost-effectiveness results: base-case.

	Totals			Incremental			ICERs	
	Cost	LYs	QALYs	Cost	LYs	QALYs	Cost per LY saved	Cost per QALY gained
Generic alendronate	$31,456	7.9007	5.9866	—	—	—	*Ref.*	*Ref.*
Denosumab	$32,334	7.9339	6.0386	$878	0.0333	0.0520	$26,389	$16,888
Zoledronate	$35,138	7.9132	6.0037	$2,804	−0.0208	−0.0350	Dominated	Dominated
Risedronate	$35,232	7.8941	5.9760	$2,899	−0.0399	−0.0626	Dominated	Dominated
Ibandronate	$35,550	7.8867	5.9663	$3,216	−0.0472	−0.0723	Dominated	Dominated
Teriparatide	$48,828	7.9308	6.0279	$16,495	−0.0031	−0.0107	Dominated	Dominated

Numbers may not add up due to rounding.

**Table 6:** 10-year risk of events: base-case.

	Hip fractures	Vertebral fractures	NHNV fractures
Denosumab	0.138	0.101	0.232
Generic alendronate	0.162	0.117	0.229
Zoledronate	0.165	0.097	0.217
Risedronate	0.170	0.115	0.227
Ibandronate	0.176	0.113	0.235
Teriparatide	0.147	0.112	0.216
