# Supplementary material for: Denosumab for Elderly Men with Osteoporosis: A Cost-Effectiveness Analysis from the US Payer Perspective
Source: J Osteoporos. 2015 Dec 9;2015:627631. doi: 10.1155/2015/627631 (PMC4689973; doi:10.1155/2015/627631)
Supplement: Supplementary file 1 — The supplementary material provides two tables with additional detail on model estimation. The first table provides 6-month discontinuation rates for all treatments, up to three years. The second table provides the relative risks of mortality for each fracture type, compared to the general population. [file 627631.f1.pdf]

Supplement

Supplemental Table 1. Discontinuation Rates

Time Point (Years)	Oral treatments*	Injectables†
0.5	27.4%	13.7%
1	29.3%	14.7%
1.5	23.7%	11.8%
2	23.0%	11.5%
2.5	30.8%	15.4%
3	25.9%	12.9%

*Includes alendronate, risedronate, and ibandronate
†Includes denosumab, teriparatide, and zoledronate

Supplemental Table 2. Relative Risk of Mortality Compared to Normal Male Population

Age	1st year after hip fracture	1st year after clinical vertebral fracture	2+ years after hip fracture	2+ years after clinical vertebral fracture	NHNV
**75-79**	5.14	5.46	4.54	3.75	5.07
**80-84**	3.91	3.73	3.45	2.56	4.06
**85-89**	2.32	2.36	2.05	1.63	2.51
**90-94**	1.49	1.62	1.21	1.12	1.62

Note: Not adjusted for comorbidities
Source: Johnell et al. 2004 [36]
